# Features and factors that dictate if terminating ribosomes cause or counteract nonsense-mediated mRNA decay

**DOI:** 10.1016/j.jbc.2022.102592

**Published:** 2022-10-13

**Authors:** Caleb M. Embree, Rabab Abu-Alhasan, Guramrit Singh

**Affiliations:** 1 Department of Molecular Genetics, The Ohio State University, Columbus, Ohio, USA; 2 Center for RNA Biology, The Ohio State University, Columbus, Ohio USA

**Keywords:** translation, ribosome, translation termination, nonsense mutations, nonsense-meditated mRNA decay, AS, alternative pre-mRNA splicing, eIF, eukaryotic translation initiation factor, EJC, exon junction complex, IP6, inositol hexakisphosphate, HTLV-1, human-T-cell leukemia virus type-1, NMD, nonsense-mediated mRNA decay, NTC, normal termination codon, PABP, poly(A)-binding protein, poly(A), polyadenylate, PRF, programmed ribosome frameshifting, PTC, premature termination codon, SR, serine–arginine, uORF, upstream ORF

## Abstract

Nonsense-mediated mRNA decay (NMD) is a quality control pathway in eukaryotes that continuously monitors mRNA transcripts to ensure truncated polypeptides are not produced. The expression of many normal mRNAs that encode full-length polypeptides is also regulated by this pathway. Such transcript surveillance by NMD is intimately linked to translation termination. When a ribosome terminates translation at a normal termination codon, NMD is not activated, and mRNA can undergo repeated rounds of translation. On the other hand, when translation termination is deemed abnormal, such as that on a premature termination codon, it leads to a series of poorly understood events involving the NMD pathway, which destabilizes the transcript. In this review, we summarize our current understanding of how the NMD machinery interfaces with the translation termination factors to initiate NMD. We also discuss a variety of *cis*-acting sequence contexts and *trans*-acting factors that can cause readthrough, ribosome reinitiation, or ribosome frameshifting at stop codons predicted to induce NMD. These alternative outcomes can lead to the ribosome translating downstream of such stop codons and hence the transcript escaping NMD. NMD escape *via* these mechanisms can have wide-ranging implications on human health, from being exploited by viruses to hijack host cell systems to being harnessed as potential therapeutic possibilities to treat genetic diseases.

Cellular life depends on the accurate decoding of genetic information into functional molecules. As instructions in mRNA are translated into proteins, ribosomes engaged in every step of translation are closely monitored to ensure that genetic information is decoded with high fidelity. For example, during translation elongation, ribosome-linked quality control mechanisms monitor for abnormally paused, stalled, or colliding ribosomes (1). These mechanisms perform the dual role of degrading problematic mRNAs and rescuing the ribosomes stuck on these mRNAs. Similarly, ribosomes undergoing translation termination are monitored by a mechanism called nonsense-mediated mRNA decay (NMD), which differentiates normal termination events from those that occur at premature termination sites. NMD serves two vital functions in the cell; it acts as a transcript quality control monitor and as a gene regulation mechanism (2). Both functions begin when translation termination is determined to be abnormal, and both result in rapid mRNA degradation. The primary difference between the quality control and gene regulation functions of NMD is if the abnormal termination arises from a mutation or a programmed feature of the transcript. In this review, we will focus on how the interplay between the ribosome, translation termination factors, and NMD machinery leads to NMD, and how certain behaviors of terminating ribosomes can sometimes suppress NMD activation.

Several scenarios can lead to stop codons that can induce NMD (3). As a quality control mechanism, NMD monitors translation termination to identify mRNAs that may have acquired premature termination codons (PTCs) because of mutations or errors during RNA biogenesis (4, 5). As the name suggests, PTCs are stop codons that occur within the normal ORF of a transcript. By rapidly degrading PTC-containing mRNAs, NMD limits the expression of truncated polypeptides, which may otherwise be detrimental to cells (6). In vertebrates, a major class of NMD substrates arises from regulated alternative pre-mRNA splicing (AS) that introduces a PTC because of a variety of AS events (*e.g.*, exon skipping or inclusion, intron retention). In this capacity, NMD serves as a gene regulatory function. NMD-targeted mRNAs also consist of normal protein coding transcripts bearing features such as upstream ORFs (uORFs), long 3′-UTRs or 3′-UTR introns, which make normal termination events resemble NMD-inducing termination events (2, 5). This group of NMD targets also includes a special set of naturally occurring mRNAs containing internal stop codons that can be recoded in a regulated manner to specify selenocysteine, the 21st amino acid (7). NMD-induced degradation of such normal mRNAs serves to fine-tune levels of encoded proteins. As NMD links abnormal translation termination to RNA degradation, it can also be employed to control levels of long noncoding RNAs and muffle pseudogene expression (6). To generalize, NMD occurs when translation termination is deemed to be abnormal. To understand why termination in a variety of contexts is sensed as abnormal by NMD, and why some such events may go undetected leading to escape from NMD, we must first establish what occurs when a ribosome terminates translation on a normal stop codon.

## Normal translation termination and ribosome recycling

### The basic translation termination reaction

**Figure 1:**
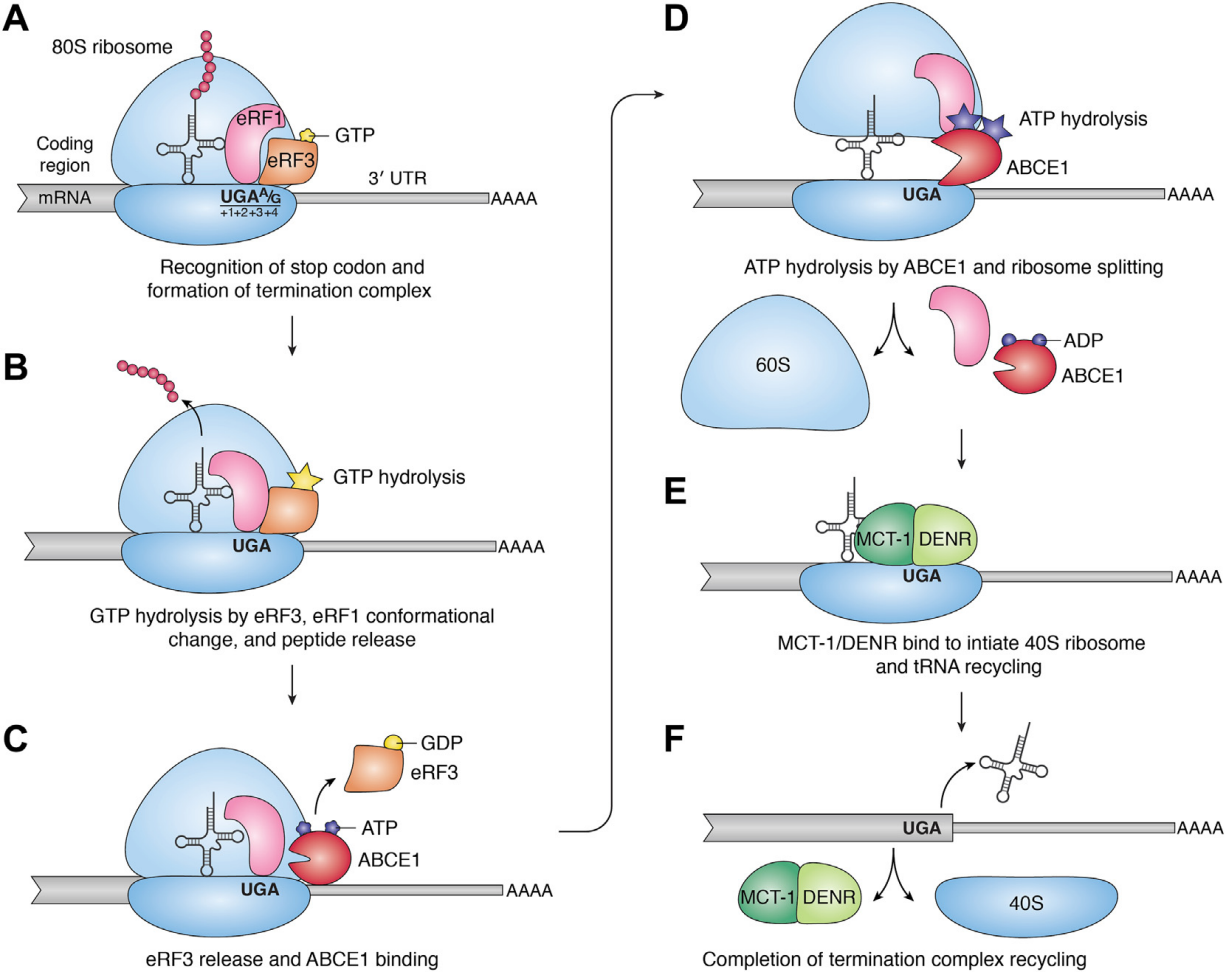
**Key steps in normal translation termination and recycling.** mRNA is shown in *gray* with the *thicker line* depicting the coding sequence and the *thinner line* depicting the “normal” 3′ UTR. The ribosome is shown in *blue*, and other termination factors are labeled. *A*, normal translation termination begins when eRF1 recognizes a termination codon (shown as UGA) and the following nucleotide (+4 position; preferred nucleotides are shown) in the A-site along with the GTP-bound form of eRF3 to form the termination complex. *B*, GTP hydrolysis by eRF3 causes a conformational change in eRF1 that positions it to mediate hydrolysis and release of the nascent polypeptide. *C*, eRF3-GDP dissociates from the termination complex, and ABCE1, bound to ATP, joins to begin ribosome recycling. *D*, ATP hydrolysis by ABCE1 leads to ribosome splitting followed by dissociation of eRF1, ABCE1, and 60S from the mRNA. *E*, the 40S recycling factor heterodimer MCT-1/DENR binds the terminated ribosome. *F*, the 40S subunit and P-site tRNA are then removed from the mRNA by MCT-1/DENR, freeing all ribosome components to engage in another round of translation.

Translation termination begins upon recognition of one of the three stop codons: UAA, UAG, or UGA (Fig. 1*A*). While sense codons are recognized by tRNAs, stop codons are recognized by eRF1 (8), a protein translation termination factor that structurally mimics tRNA. Like tRNAs, eRF1 enters the empty amino-acyl (A)-site on the ribosome where it forms hydrogen bonds with stop codons on mRNA (8, 9). eRF1 binding to the A-site is just as rapid as aminoacyl-tRNA binding, but eRF1 appears to reside in this position in the ribosome for a longer period (10). Importantly, unlike sense codon recognition, where tRNA pairing is limited to the triplet nucleotides of a codon, the nucleotide (nt) following a stop codon (+4 nt) plays a key role in the termination reaction (Fig. 1*A*) (11). After recognition of the stop codon by eRF1 within the ribosome decoding center, the mRNA is compacted and the +4 nt is pulled into the A-site (8, 12). This mRNA conformation results from the flipping of the 18S rRNA adenine (A) base at position 1825 (rRNA numbering is as in Ref. (8) based on rabbit/mammalian 18S rRNA sequences), which stacks with the +2 nt of the stop codon, which in turn stacks with the +3 nt. These base interactions further allow G626 and C1698 of the rRNA to stack with +4 and +5 nt of the mRNA, respectively, thereby extending the recognition beyond the triplet stop codon (8, 12). Importantly, stacking between rRNA and mRNA is more stable with a purine in the +4 nt position (8), which could explain why purines are common in the +4 position, with UAA(A/G) and UGA(A/G) being the two most common tetra-nt stop codons in eukaryotes (13). Similarly, a purine at the +5 position can strengthen a weak stop with pyrimidine at the +4 position (11). Thus, translation termination is dictated by interactions beyond eRF1–stop codon recognition and includes mRNA–rRNA interactions.

eRF1 does not act alone at the terminating ribosome; it functions in tandem with the GTPase eRF3 (14). In humans, two genes, *GSPT1* and *GSPT2*, encode translation termination GTPases eRF3a and eRF3b, respectively (15). eRF3a is ubiquitously expressed and considered the predominant source of eRF3 activity, whereas eRF3b expression in most human tissues is lower, with higher expression in certain tissues such as the brain and testis where it may have a more specialized function (16). We will generically refer to these two GTPases as eRF3. Binding of eRF1 to eRF3 stabilizes the GTP-bound form of eRF3 (Fig. 1) (17, 18). The eRF1–eRF3–GTP complex can either form by itself or assemble in association with the ribosome (19, 20, 21). The eRF1–eRF3–GTP complex then binds the A-site of the terminating ribosome much faster than eRF1 can alone (10, 22). Together, the ribosome, eRF1, and GTP-bound eRF3 form the termination complex (23). Stop codon recognition by eRF1 accelerates GTP hydrolysis by eRF3 (20), and the GTP hydrolysis function of eRF3 accelerates its release from the complex (10). Subsequently, a conformational change occurs in eRF1, whereby its catalytic GGQ motif is accommodated in the peptidyl transferase center of the ribosome. eRF1 thus positioned catalyzes the hydrolysis of the ester linkage between the tRNA and the nascent polypeptide, releasing it from the peptidyl (P) site of the terminating ribosome (Fig. 1*B*) (8, 12, 19, 20). The hydrolysis reaction favors the release of eRF1 although it is energetically costly. Subsequently, eRF1 affinity to the terminated ribosome is substantially decreased (10).

### Enhancers of translation termination

Several other *trans*-acting factors can further promote the termination activity of eRF1 and eRF3, chief among these is the polyadenylate (poly(A))-binding protein (PABP), which binds to the poly(A) tails at the 3′ ends of mRNA. A C-terminal domain in PABP binds with the PAM2 motif in the N terminus of eRF3 (24). It was observed using *in vitro* reconstitution assays that under low release factor concentrations, eRF3–PABP interaction enhances the termination reaction by stabilizing the binding of eRF3 to the ribosomal complexes, which leads to stimulation of stop codon recognition and peptidyl-tRNA hydrolysis (24). The effect of 3′ poly(A)-tail–bound PABP on termination may also be maximal when stop codons occur close to poly(A) tails (25, 26, 27, 28, 29) (also see later). Interestingly, the stimulating effect of PABP on termination *in vitro* is seen both with and without its binding to the poly(A) tail, which suggests that PABP may enhance termination *via* multiple mechanisms (24). One such mechanism could be through interaction between PABP and translation initiation factor eukaryotic translation initiation factor 4G (eIF4G), a connection that can suppress premature termination and promote normal termination and ribosome recycling (30, 31) While PABP can enhance translation termination *in vitro* under certain conditions, it remains to be seen if PABP plays a similar role in translation termination *in vivo*.

In addition to PABP, other factors are known to enhance the termination reaction. Elongation factor eIF5A, which binds to the ribosome such that it partially occupies the empty exit (E)-site of the elongating ribosome to stabilize peptidyl-tRNA for peptide bond formation, also promotes peptidyl-tRNA hydrolysis by eRF1 (32) and eRF1 release (10), thereby enhancing the termination reaction (32, 33). Dbp5, a DEAD-box ATPase involved in mRNA export, and its activator Gle1 and inositol hexakisphosphate (IP6), have also been implicated in translation termination in yeast (34, 35, 36). Dbp5 and Gle1 physically interact with termination factors, enhance eRF3 recruitment to the ribosome, and the loss of Dbp5, Gle1, or IP6 production increases stop codon readthrough (34, 35). DDX19B, the Dbp5 homolog in humans, increases the speed of termination by enhancing peptidyl-tRNA hydrolysis by binding to eRF1 (37, 38). NMD-activating UPF proteins can modulate premature termination (see later), but it is not clear if they also act on normal termination.

### Ribosome recycling

Following nascent polypeptide hydrolysis, eRF3 bound to GDP is released from the termination complex. Ribosome recycling begins as the highly conserved recycling factor ABCE1 (Rli1 in yeast), an iron–sulfur cluster–containing ATPase, binds to eRF1 at the same domain that was earlier occupied by eRF3 (Fig. 1*C*) (8, 21). ATP hydrolysis by ABCE1 promotes the separation of the 60S ribosome subunit from the 40S subunit, which remains bound to tRNA and mRNA (Fig. 1*D*) (39). This first step in ribosome recycling is also dependent on eIF3j/Hcr1, which may act by either recruiting ABCE1 to the termination complex or by stimulating its ATPase activity (40, 41). Following the release of the 60S subunit by ABCE1/eIF3j, the remaining 40S subunit–deacylated tRNA–mRNA complex is disassembled and recycled by the MCT-1/DENR heterodimer (Tma20/Tma22 in yeast) (Fig. 1, *E* and *F*) (42). This 40S subunit recycling activity is also shared by eIF2D (Tma64 in yeast) (41, 42, 43), whose N and C terminus are homologous to MCT-1 and DENR, respectively (44). This process of ribosome recycling is not only critical in ensuring that the ribosome does not resume translation in the 3′ UTR but also to maintain the pool of free ribosome subunits for another round of translation.

## Abnormal translation termination triggers NMD

When a ribosome terminates translation at a normal termination codon (NTC) at the end of a conserved ORF, it is often followed by several subsequent rounds of translation. However, if the ribosome terminates translation prematurely within an ORF, for example, because of the presence of a PTC, it results in translation repression (45, 46) and rapid mRNA destabilization (2, 5, 6). Next, we summarize our understanding of transcript features and molecular interactions between translation termination and NMD factors that divert the mRNAs to the path of NMD.

### Transcript features that distinguish normal and NMD-inducing stop codons

The signals that initiate NMD following translation termination at certain stop codons can be categorized into two general mechanistic themes.

#### Abnormally long 3′ UTRs

**Figure 2:**
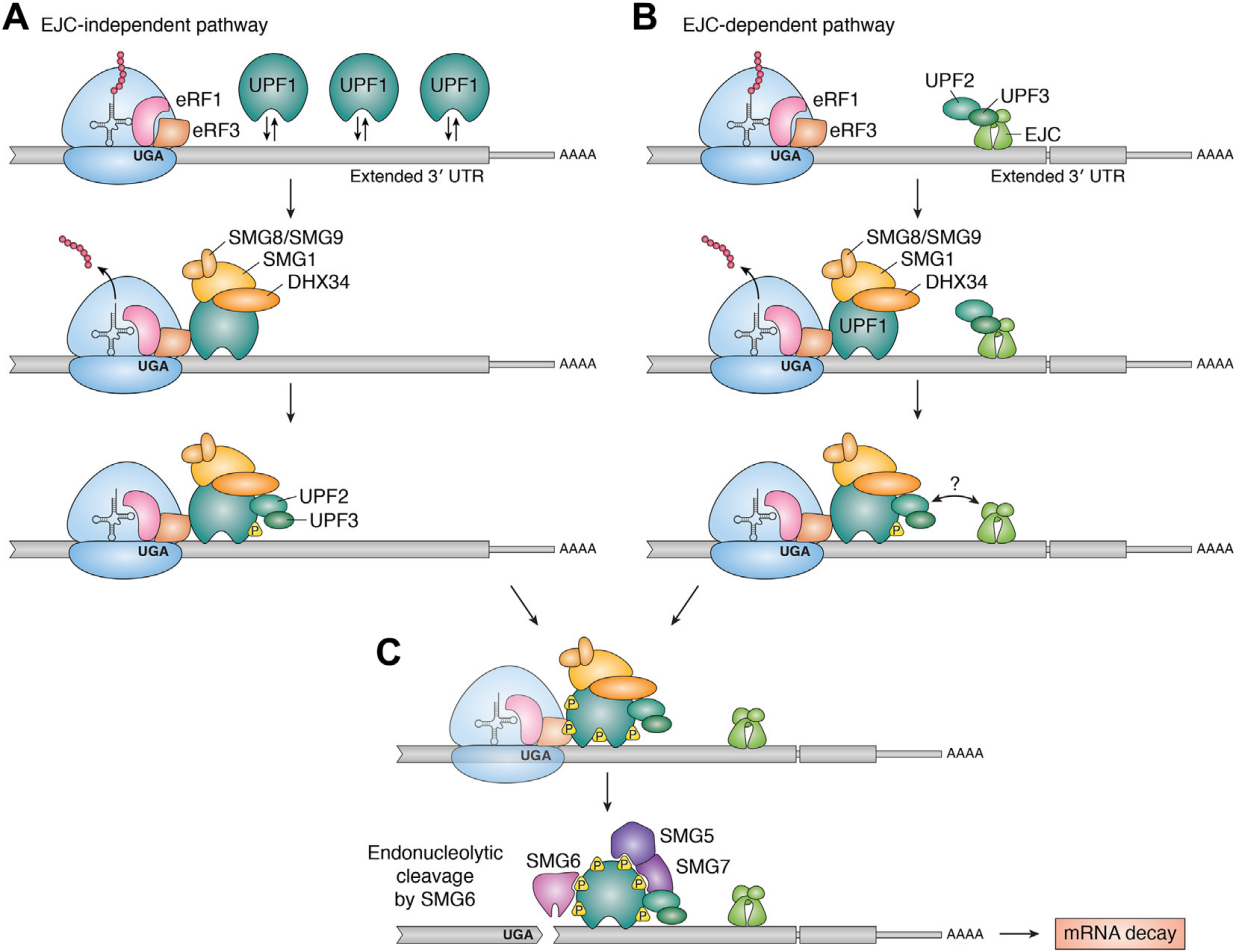
**NMD can occur in an EJC-dependent or EJC-independent manner.** mRNA is shown in *gray*. The introduction of premature termination codon in coding sequence (*thicker line*) results in an “extended 3' UTR” that also includes the normal 3′ UTR (*thinner line*). The ribosome is shown in *blue*, and other important proteins are labeled. Recognition of NMD-inducing translation termination codon occurs through one of two general pathways: the EJC-independent (*A*) or EJC-dependent (*B*) pathway. *A*, the EJC-independent pathway begins when UPF1, the core NMD factor, is bound downstream of the terminating ribosome at abnormally high levels. After interaction between UPF1 and the termination complex, other NMD factors are recruited to assemble the “SURF” complex, which includes UPF1 phosphorylation factors SMG1 (the kinase) and its regulatory SMG8/9 heterodimer. This complex may include or subsequently recruit DHX34, an RNA helicase that may aid in interaction of UPF1 with its activators UPF2 and UPF3B (or its paralog UPF3A). Formation of the SURF complex leads to UPF1 phosphorylation (represented by *yellow triangles* labeled “P”) by SMG1. *B*, the EJC-dependent NMD requires the presence of an EJC downstream of the terminating ribosome that enhances NMD by recruiting UPF2 and UPF3B (or UPF3A) to mRNA. Following assembly of the SURF complex at the terminating ribosome and UPF1 interaction with its activators UPF2 and UPF3B (or UPF3A), which can be aided by DHX34, UPF1 gets phosphorylated by SMG1. *C*, after initial phosphorylation, UPF1 is phosphorylated more extensively. It is unknown how long the ribosome and release factors remain associated with the phosphorylated UPF1 (indicated by *faded shapes*). The extensive phosphorylation of UPF1 leads to the recruitment of SMG6 and SMG5/7 heterodimer, which initiate mRNA degradation *via* multiple mechanisms including SMG6-catalyzed mRNA endonucleolytic cleavage close to termination codons. EJC, exon junction complex; NMD, nonsense-mediated mRNA decay.

The occurrence of early stop codons (*e.g.*, PTC, uORF stop codon) can turn the downstream coding sequence into a UTR, thereby extending the 3' UTR. According to current NMD models, when translation termination occurs in an altered 3′ UTR context, it can be recognized as abnormal because of multiple non–mutually exclusive reasons (25, 47, 48). One reason could be the diminished stimulation of termination by PABP. When termination happens far upstream of the normal 3' UTR, the increased distance between the terminating ribosome and the poly(A) tail could reduce or compromise interaction between eRFs and PABP (24, 26, 27, 28, 30). Another basis for extended 3′ UTRs to promote abnormal termination and NMD is by increasing the RNA occupancy of central NMD factor UPF1, which binds RNA before translation in a length-dependent and sequence-independent manner (49, 50, 51). The translating ribosome is thought to remove any bound UPF1 in its path, thereby reducing UPF1–mRNA binding when translation terminates in a normal context (49). When translation terminates in an abnormal context, such as that of a PTC, it can lead to a higher local concentration and/or more likelihood of UPF1 binding to the extended 3' UTRs (Fig. 2*A*). Such transcript-bound UPF1, through its interactions with the 40S ribosome (52) as well as with eRF1 and eRF3 (26, 53, 54), can engage with termination machinery to trigger NMD (25, 49, 55). The window of opportunity for UPF1 to interact with release factors and cause premature termination can further be increased because of reduced engagement between eRFs and PABP. Interestingly, normal error-free mRNAs with abnormally long 3′ UTRs (>800 nt in humans) can also be destabilized by UPF1 (50), possibly because of termination on these mRNAs deemed to be abnormal as a result of higher UPF1 occupancy in 3′ UTRs.

#### 3′ UTR-bound proteins

Another major reason for a translation termination event to be identified as abnormal is the presence of UPF1 activating factors in 3' UTRs. The most prominent example is the exon junction complex (EJC), which is deposited upstream of mRNA exon–exon junctions during mRNA splicing (55, 56, 57) and is disassembled by the ribosome during translation (57, 58). Generally, NTCs occur in the last exon, so all EJCs are removed before translation terminates. However, if a stop codon occurs sufficiently upstream of the last exon–exon junction such that at least one EJC remains bound in the 3′ UTR, abnormal termination and NMD ensues (Fig. 2*B*) (2). The presence of at least one exon–exon junction, and hence the EJC, downstream of a stop codon is one of the strongest predictors of NMD susceptibility for human mRNAs (48). Conversely, if translation terminates either in the last exon or close to the last exon–exon junction in the penultimate exon, the mRNA evades NMD possibly because of removal of the EJC from the last exon junction (48). Current NMD models suggest that terminating ribosome-bound UPF1 senses downstream EJCs through bridging interactions mediated by UPF2 and UPF3 proteins (26, 59, 60, 61). However, recent evidence that human UPF3 paralogs UPF3A and UPF3B can activate NMD even when they lack EJC binding ability suggests that interaction between EJC and UPF3 proteins is not necessary for premature termination (62, 63). It is possible that another mode of communication exists between the terminating ribosome and the downstream EJC (62, 63). One possible function of 3' UTR-bound EJCs could be to recruit UPF2 and UPF3 proteins to 3′ UTRs to increase their likelihood of UPF1 activation (Fig. 2*B*) (55, 60, 61, 64, 65). Importantly, some genes naturally contain introns in their 3' UTRs, which causes normal termination events on their mRNAs to resemble abnormal termination because of the presence of a downstream EJC (66, 67). Thus, the presence of an EJC in the 3′ UTR is a potent trigger for abnormal termination and NMD, but how EJC enhances these processes remains far from clearly understood.

In addition to the EJC, the serine–arginine (SR) proteins, which are also recruited to RNA during its biogenesis in the nucleus (68), can also enhance NMD (69, 70) possibly *via* their binding to 3' UTRs. SRSF1, a prototypical SR protein, has been suggested to interact directly with UPF1 and activate NMD when present in 3′ UTRs (71). Another SR protein, SRSF2, can promote NMD by enhancing EJC deposition/binding on RNA (72). It is possible that other SR proteins including SRSF1 can also act in NMD *via* the EJC. All 12 canonical SR proteins and many SR-like proteins are physically associated with the EJCs purified from human cells with SR protein binding sites enriched in the vicinity of the EJC (73, 74).

Regardless of the signal that induces NMD, once termination is determined to be abnormal, subsequent steps in the NMD pathway are set in motion that are summarized in Figure 2. For a more detailed discussion of the modes of NMD initiation as well as the resulting mRNA degradation mechanisms, we refer the readers to excellent recent reviews (2, 75, 76).

### Interactions between UPF proteins and termination machinery

Following the recruitment of NMD factors by one of the NMD-inducing signals, the core NMD factors engage with the terminating ribosome and associated release factors, and possibly even with ribosome recycling machinery. The precise mechanistic details of how NMD factors influence and alter events during termination to initiate NMD remain obscure. However, some details of the interactions between NMD and termination/recycling machinery have emerged, mainly from work in yeast and mammalian cell culture models. Notably, there are several discrepancies in the observed interactions and roles of the UPF factors during translation termination. Such differences could stem from different model systems and experimental approaches employed or from our incomplete understanding of the players and interactions between NMD and translation termination factors.

#### Evidence from yeast

In *Saccharomyces cerevisiae*, all three UPF proteins have been shown to interact with ribosomes (77, 78, 79, 80). This interaction happens on polysomes (77, 79, 80), where the mRNA degradation phase of NMD is also reported to occur (81, 82). Such interactions can occur because of the direct binding of UPF factors to ribosomes or because of UPF interactions with release/recycling factors. Selective profiling of footprints of ribosomes bound to each of the three yeast UPF proteins shows that detection of UPF–ribosome complexes progressively increases toward the 3′ end of coding sequences (80). These UPF factor–ribosome complexes are slightly more enriched on NMD-targeted transcripts as compared with nontargets. This finding suggests that unlike in human cells where translocating ribosomes strip UPF1 from RNA (49), translating ribosomes in yeast cells do not remove UPF1 from mRNAs. Alternatively, it is possible that the UPF1–ribosome complex formation is unaffected by the dissociation of mRNA-bound UPF1 by translocating ribosomes. Nonetheless, this new study suggests that UPF proteins can engage with ribosomes before they reach annotated stop codons (80). While it is not known if these are elongating or terminating ribosomes, these observations suggest that the NMD machinery may continuously monitor the termination status of ribosomes as they progress along a coding sequence.

**Figure 3:**
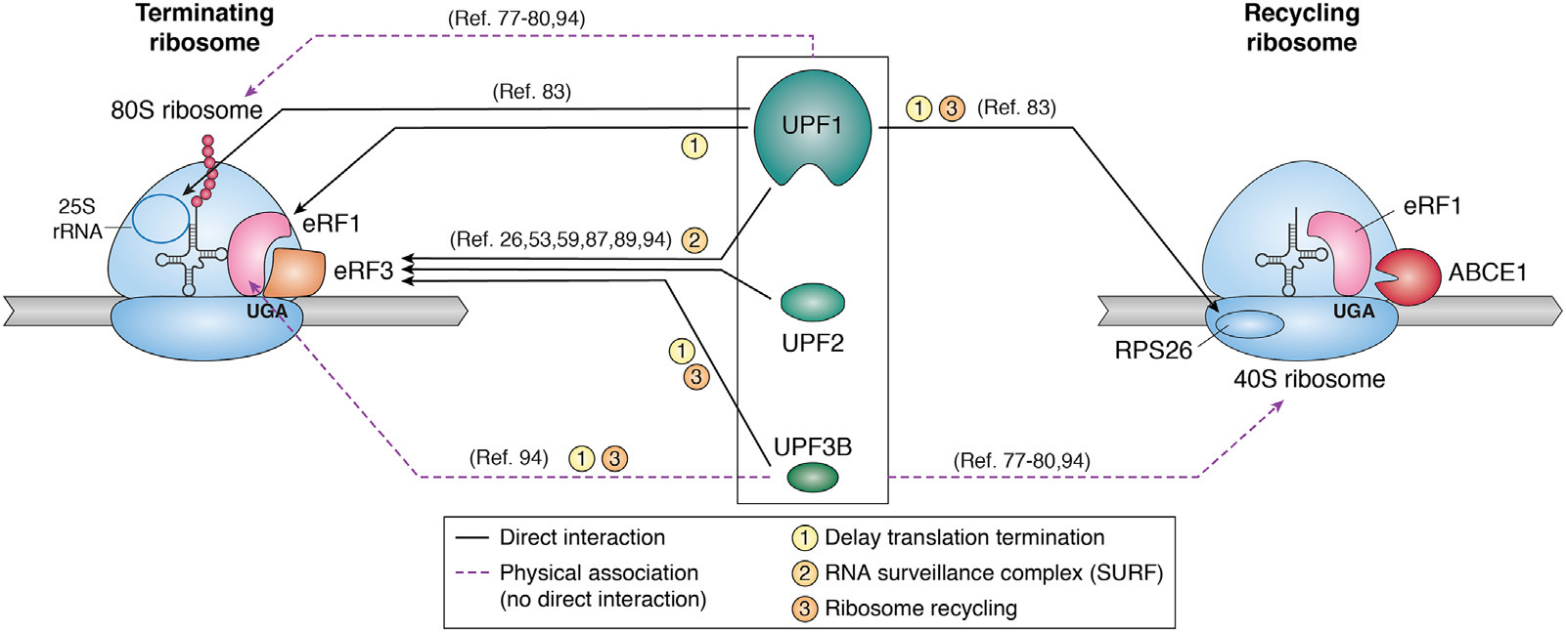
**Interactions between UPF proteins and termination machinery.***Left*, a terminating ribosome. *Right*, a recycling ribosome. The UPF proteins interact with the ribosome and termination factors in both a direct (*black lines*) and indirect (*purple lines*) fashion. All three UPF proteins can interact with the 80S ribosome and the 40S subunit although the specific contacts within these assemblies are known only for UPF1. All three UPF proteins are also reported to interact with termination factors. The numbers on each *arrow* represent possible functions of each interaction listed in the legend below. References that provide evidence for the interactions between UPF proteins and termination machinery are included in parentheticals.

How UPF proteins interact with ribosomes is best understood in the case of UPF1. In an *in vitro* system, recombinant UPF1 can directly bind to 80S ribosomes by binding to the L1 stalk of the 25S rRNA (Fig. 3) (83). This binding positions UPF1 at the ribosomal E site of tRNA-bound ribosomes. Interestingly, UPF1 has also been shown to interact with the 40S ribosomal subunit *via* a direct interaction with Rps26 (Fig. 3) (84). While the contribution of UPF1–ribosome interactions to its function in NMD is yet to be completely understood, the UPF1–40S ribosome interaction may aid in ribosome recycling following termination at PTCs, as loss of UPF1 impairs this process (52). Notably, the role of UPF1 in ribosome recycling at PTCs is also dependent on its ATP hydrolysis activity and UPF2 binding ability (85).

Early studies in yeast revealed a genetic interaction between UPF factors and translation termination. In yeast cells lacking UPF proteins, termination at PTCs was inefficient such that readthrough of translation was observed at a much higher rate (86, 87). Such an effect was most pronounced for UPF1 as compared with UPF2 and UPF3, indicating a more direct role of UPF1 in termination. The role of UPF2 and UPF3 in nonsense suppression was suggested to be more of regulatory nature and independent of their function in inducing rapid decay of PTC-containing mRNAs. Importantly, inhibition of mRNA decay by compromising mRNA decapping or 5′-3′ exonucleolytic decay did not result in increased nonsense suppression (75). Another study using bicistronic reporters for monitoring readthrough at nonsense codons did not find a role for UPF proteins in this process, suggesting that UPF function in nonsense suppression could be context dependent (88).

The role for UPF proteins in termination is further supported by physical interactions between all three yeast UPF proteins and eRF1 and eRF3 in cell extracts as well as when produced recombinantly *in vitro* (Fig. 3) (87, 89). UPF1 can bind simultaneously to eRF3, UPF2, and UPF3, whereas UPF2 and UPF3 compete with eRF1 to bind to eRF3 (87). Furthermore, UPF1 RNA-binding activity antagonizes its interaction with eRF3 (89). Interestingly, UPF1 function in nonsense suppression and mRNA decay could be separated by mutations impairing its various biochemical activities. A UPF1 mutant that could bind ATP but not hydrolyze it was still able to function in nonsense suppression but was not able to induce rapid mRNA decay (90). On the other hand, a UPF1 mutant with reduced UPF2 binding could not support nonsense suppression even though it was able to destabilize PTC-containing mRNAs (91). Taken together, these data from early work in yeast hinted that NMD-inducing termination is comprised of a series of steps that involve interplay between UPF factors and the terminating ribosome, but molecular details of this process remain largely a mystery.

#### Evidence from mammalian cells

In mammalian cells, like in yeast, UPF proteins are also detected on polysomes (92, 93). Furthermore, human UPF1, UPF2, and UPF3B can bind to the 80S ribosome, individually and as a complex (Fig. 3) (94). However, it remains to be seen if the UPF1 interaction with the ribosome is direct, through 25S rRNA or Rps26 as reported in yeast (84). Regarding UPF interactions with release factors in mammalian/human cells, there are conflicting data in the literature. Early studies that focused only on UPF1 showed that it can interact with both eRF1 and eRF3, binding to the GTPase domain of eRF3 (26, 53, 89). Similarly, it was found that UPF2 can also interact with eRF3 to further activate UPF1 within the SURF complex (59). The UPF2–eRF3 interaction occurs in opposition to the UPF2 interaction with UPF3B, though the UPF2–UPF3B interaction is stronger (59).

A more recent investigation compared interactions of the three human UPF proteins with termination factors. Both UPF1 and UPF3B were found to interact strongly with release factors in human cell extracts, whereas UPF2 binding to release factors was weaker (Fig. 3) (94). However, only UPF3B was found to influence translation termination in a reconstituted system *in vitro*. Depending on the stage of translation termination, UPF3B was suggested to have two opposing functions (94). Before stop codon recognition, particularly if release factors are limiting, UPF3B can prevent translation termination (94). After termination, UPF3B can promote ribosome recycling (94). Both of these UPF3B functions are antagonized by UPF2 (94), suggesting that function of UPF3B during termination is distinct from its role in NMD activation together with UPF2. The dual roles of UPF3B in translation termination were attributed to the direct interactions between UPF3B and release factors, although the stronger eRF3–UPF3B interaction may stabilize the interaction between UPF3B and eRF1, allowing the three proteins to form a complex (94). It was suggested that before stop codon recognition, the interaction between UPF3B and eRF3 leads to sequestration of the release factors, delaying translation termination (94). Subsequently, after the first step of translation termination occurs, the interaction between UPF3B and release factors is proposed to promote ribosome recycling (94). Whether these UPF3B functions in translation termination also hold true in living cells remains to be seen.

Notably, vertebrate genomes also encode a UPF3B paralog, UPF3A, which is ubiquitously expressed but enriched in select tissues such as the testis and brain (95). The available evidence suggests that UPF3A has weak NMD activation capability but can also antagonize the ability of UPF3B to activate NMD in certain contexts (62, 63, 96, 97, 98). Interestingly, the NMD activation potential of UPF3A becomes comparable to that of UPF3B when a poorly characterized region of the protein that lies within the UPF2- and EJC-binding domains is replaced with an equivalent region of UPF3B (62). It is noteworthy that this “mid-domain” of UPF3B was shown to directly interact with eRF3 (94) and bears point mutations linked to human neurodevelopmental disorders (99). How UPF3A contributes toward overall “UPF3” function during termination remains an important question.

Most of the research into human NMD has focused on one specific isoform of UPF1. Recently, another UPF1 isoform possessing a longer regulatory loop and greater affinity for RNA was described (100). This isoform regulates a different subset of NMD targets than the more widely studied UPF1 isoform (100). It is unknown if this long loop UPF1 isoform interacts with the termination machinery in the same manner as the short loop isoform. It is also possible that the increased RNA affinity of the UPF1 long loop isoform may influence some of the events that inhibit traditional NMD, as discussed later in this review.

### Is NMD-inducing termination slower than normal termination?

An important question in the NMD field is if the translation termination reaction itself is different on NMD-inducing stops as compared with the normal stops. Studies that have compared termination at these two types of stop codons have arrived at conflicting conclusions. Estimation of ribosome occupancy/dwell time in yeast extracts showed that, as compared with normal stops, translation termination on NMD-inducing stop codons yields distinct toeprints suggesting that ribosomes persist longer on NMD stops, perhaps because of inefficient/slow termination (28). Such ribosomes were also capable of backtracking and reinitiating translation at nearby upstream start codons. This “aberrant” behavior of terminating ribosomes was abolished in extracts lacking UPF1 or when either normal 3′ UTR or PABP was positioned in the immediate vicinity of the stop codons (28). Similar ribosome toeprints, diagnostic of slow ribosome release from NMD-inducing stop codons, were also observed on human β-globin mRNA in rabbit reticulocyte lysates (101). These earlier observations argued that NMD-inducing termination reactions are slow and aberrant and hence different from normal termination. However, a more recent study in human cells did not detect differences in ribosome occupancy at NMD-inducing and normal stop codons either in *in vitro* translation extracts or using ribosome profiling to probe ribosome occupancy *in vivo* (102). While the differences in ribosome occupancy at NMD-inducing stops in human extracts and rabbit reticulocyte lysates were attributed to an mRNA cleavage activity giving rise to ribosome toeprints in reticulocyte lysates (102), discrepancies between the yeast and human studies remain unexplained. Thus, similarities or differences between the termination reaction at normal *versus* NMD-inducing stop codons remain unresolved.

One consequence of prolonged ribosome residence on stop codons because of inefficient or slow termination (or ribosome recycling) will be collisions between the terminating (or recycling ribosome) and the trailing ribosome. Ribosome collisions have been well documented to occur when an elongating ribosome runs into another stalled elongating ribosome (103, 104, 105). There is some evidence suggesting that an elongating ribosome can also collide with a terminating ribosome, most frequently if termination is prolonged or inefficient. In mammalian cells expressing the dominant negative form of the release factor eRF1^AAQ^, which arrests translation termination, the ribosome collision sensor ZNF598 is found to associate with the collided ribosomes at stop codons (104, 106). Furthermore, in both yeast and human cells, the footprints of collided ribosomes, termed a disome, are enriched where the A-site of the leading ribosome is located at a stop codon (107, 108). It remains unknown if ribosome collisions occur more frequently at the NMD-inducing stop codons as compared with normal stop codons, as would be expected if NMD-inducing termination was slower. What is now known, however, is that the ribosome collision–sensing proteins ZNF598 and EDF1 are dispensable for NMD as their depletion in mammalian cells did not affect the decay of NMD reporters or endogenous NMD targets (46, 109). It is possible that collisions at stop codons are sensed by yet another mechanism, or such collisions are inconsequential for NMD. On the other hand, NMD factors may be important to ensure efficient translation termination at NMD-inducing stop codons, and their depletion can potentially cause termination arrest leading to ribosome collisions. This has been observed in human cells on an NMD reporter following NMD arrest *via* pharmacological inhibition of the UPF1 kinase, SMG1 (46). The role of ribosome collisions in NMD and the contributions of NMD factors in responding to ribosome collisions remain important avenues for future investigations.

In summary, despite the progress outlined in the preceeding paragraphs, the picture of how UPF proteins engage with translation termination machinery during NMD and how the NMD-inducing termination reaction proceeds remains incomplete in any system. To fill the remaining gaps, we need to understand if the various interactions of UPF factors with the ribosome, termination factors, and with mRNA and mRNA-binding proteins occur in a particular ordered fashion or if these events occur in a more stochastic manner to initiate NMD. A deeper understanding of the kinetics of normal *versus* NMD-inducing termination reaction is also needed along with the appreciation of how these events are influenced by the NMD factors. Some of these key outstanding questions can be resolved through the application of new tools and technologies that have recently illuminated the biophysical underpinnings and provided an atomic-level view of the basic steps of normal translation termination (8, 9, 10). In addition, a single-molecule microscopy–based direct visualization of NMD induction at an individual mRNA in live cells is now possible (110). This work has revealed that not every termination event at a PTC leads to NMD and has opened the door to further dissect NMD mechanisms inside cells.

## Alternative outcomes during elongation and termination that can lead to NMD activation or escape

As the ribosome transitions through the various states of each elongation cycle, occasional unexpected outcomes can lead to recoding of the message (111). For example, an elongating ribosome can shift into a different reading frame, which will not only change the sequence of the encoded polypeptide but also change the termination context and thereby alter mRNA stability *via* NMD. Similarly, a terminating ribosome can take alternative routes that may result in continued translation. For example, during the termination reaction, eRF1 may be outcompeted by a noncognate aminoacyl-tRNA for recognition of the stop codon, causing the ribosome to readthrough the stop codon and continue translation. If such an event occurs at a PTC, a consequence can be a full-length polypeptide and failure to induce NMD. As discussed later, these variations in outcomes during translation are dictated by local mRNA sequence and structural features. In addition, certain locations within an ORF (*e.g.*, close to the start codon) are more prone to alternative outcomes that override termination and cause an escape from NMD (112, 113). In the following sections, we discuss several examples of events during translation that unexpectedly cause or counteract NMD that have emerged from decades of work on mechanisms of translation and NMD, particularly of mRNAs bearing nonsense mutations that cause disease. Importantly, following ribosome frameshifting or readthrough, the eventual NMD outcome will be dictated by the location of the final stop codon and its impact on the NMD-inducing signals, the 3' UTR-bound NMD activators, and/or the 3′ UTR length.

### Ribosome frameshifting during elongation can lead to NMD

Ribosome frameshifting occurs when a translating ribosome moves out of its normal reading frame on mRNA and continues translation in a new reading frame. Such a shift in ribosome reading frame can occur because of an active mechanism, referred to as programmed ribosome frameshifting (PRF), which causes ribosomes to “slip” upstream or downstream, mostly by one nucleotide (114). Ribosomes can also move into a new reading frame because of a change in the protein-coding sequence by a length that is not a multiple of 3, either because of insertion/deletion of nucleotides (frameshift mutations) or because of AS. Regardless of the cause of ribosome frameshifting, all such events have the potential to result in an NMD-inducing stop codon (4).

#### Ribosome frameshifting caused by viral sequences

PRF can occur because of certain special mRNA sequence features. The most prominent and widespread signal for PRF consists of mRNA structural elements such as a pseudoknot or a stem–loop that are connected by a spacer element to an upstream “slippery” sequence (114). Such a tripartite PRF signal is common in genomes of many RNA viruses (115, 116). Having multiple ORFs in the same transcript staggered in different reading frames allows viruses to compact more genetic information into a smaller genome (116). While PRF is often used to produce multiple products from one transcript, it can also be used as a regulatory mechanism, as a frameshift in the reading frame would introduce a PTC and induce NMD (117, 118). The degree of transcript instability caused *via* NMD by programmed ribosomal frameshifting is a function of the strength of the frameshifting signal and mRNA secondary structure to slow the translating ribosomes (119).

As a −1PRF will destabilize viral transcripts *via* NMD, many viruses have developed mechanisms to protect their transcripts from NMD while producing proteins *via* frameshifting. Such is the case of human-T-cell leukemia virus type-1 (HTLV-1), the virus responsible for adult T-cell leukemia (120). Reporter RNAs based on HTLV-1 genome are sensitive to NMD as a result of PRF (120). Furthermore, the HTLV-1-encoded Rex protein blocks NMD of these reporters, suggesting a mechanism for HTLV-1 to reduce the NMD sensitivity of its genomic RNA (120). The use of PRF and mechanisms to avoid NMD as a result are not unique to viruses that infect humans. The plant infecting turnip crinkle virus employs a different method to avoid NMD (121). Downstream of the termination codon in the turnip crinkle virus genomic RNA is an unstructured region of RNA that reduces NMD efficiency (121). Mechanisms that viruses use to avoid ribosomal frameshifting–triggered NMD are a potential therapeutic target for treating diseases caused by viruses.

#### Ribosomal frameshifting signals in eukaryotic mRNAs

Ribosomal frameshifting linked to NMD has also been co-opted as a gene regulatory method in eukaryotes. In *S. cerevisiae* many genes encoding proteins associated with telomeres have programmed frameshifting signals (119). The programmed frameshift is used to maintain the proper stochiometric ratio between chromosomes and telomerases by degrading transcripts *via* NMD when more telomerase is not needed (119). When the UPF proteins are knocked out in yeast, their telomeres are shortened, which in turn shortens cellular life span (122). Previously, human *CCR5* mRNA was shown to undergo programmed ribosomal frameshift that introduces a PTC and subjects the mRNA to NMD (123). However, a recent re-evaluation of the frameshift reporters from the original study have disputed the occurrence of programmed ribosomal frameshifting in *CCR5* mRNA (124). Therefore, even though some examples exist of mammalian genes containing retrovirus-derived elements that induce -1PRF (125, 126, 127), but as of yet, there is no evidence of -1PRF signals in mammalian genes that are not derived from retroelements.

In addition to PRF, other sequence features in coding sequences can increase the frequency of frameshifting by the elongating ribosome, thereby potentially inducing NMD. One such feature is poly(A) tracts within the mRNA coding region that result from repetitive lysine codons. While translating poly(A) tracts, ribosomes “slide” on these sequences, shift into a new reading frame, and can lead to premature termination followed by NMD, a phenomenon that has been observed both in yeast and human cells (128, 129). Notably, the ribosome sliding behavior and resulting mRNA suppression by NMD is the strongest on uninterrupted A stretches and is weaker on polylysine-encoding stretches that include AAG as lysine codons. The connection between poor elongation and ribosome frameshifting to induce NMD apparently also extends to coding sequences where suboptimal codons are more frequent. An investigation of global NMD targets in yeast found that mRNAs that are otherwise normal looking but have a preference for nonoptimal codons are targeted to NMD (4). A higher rate of out-of-frame translation observed on these mRNAs suggests that translation of nonoptimal codons is likely to induce ribosome frameshifting, thereby causing premature termination and NMD. It remains to be seen whether similar mRNA features can subject mRNAs to NMD in organisms other than *S. cerevisiae*.

When frameshift mutations force a ribosome into a new reading frame, NMD can also ensue when translation terminates in this new reading frame, provided such termination occurs in a context that deems it to be abnormal. Importantly, the basic NMD-inducing contexts discussed previously (*e.g.*, EJC in 3′ UTR) are further governed by features such as the sequence surrounding PTCs, and the location of PTCs in relation to the ORF, which are discussed in more detail later. Nevertheless, whether frameshifting mutations trigger or escape NMD has an important bearing on gene expression from the mutated allele, which can provide new therapeutic avenues (130).

### Stop codon readthrough can mute NMD

**Figure 4:**
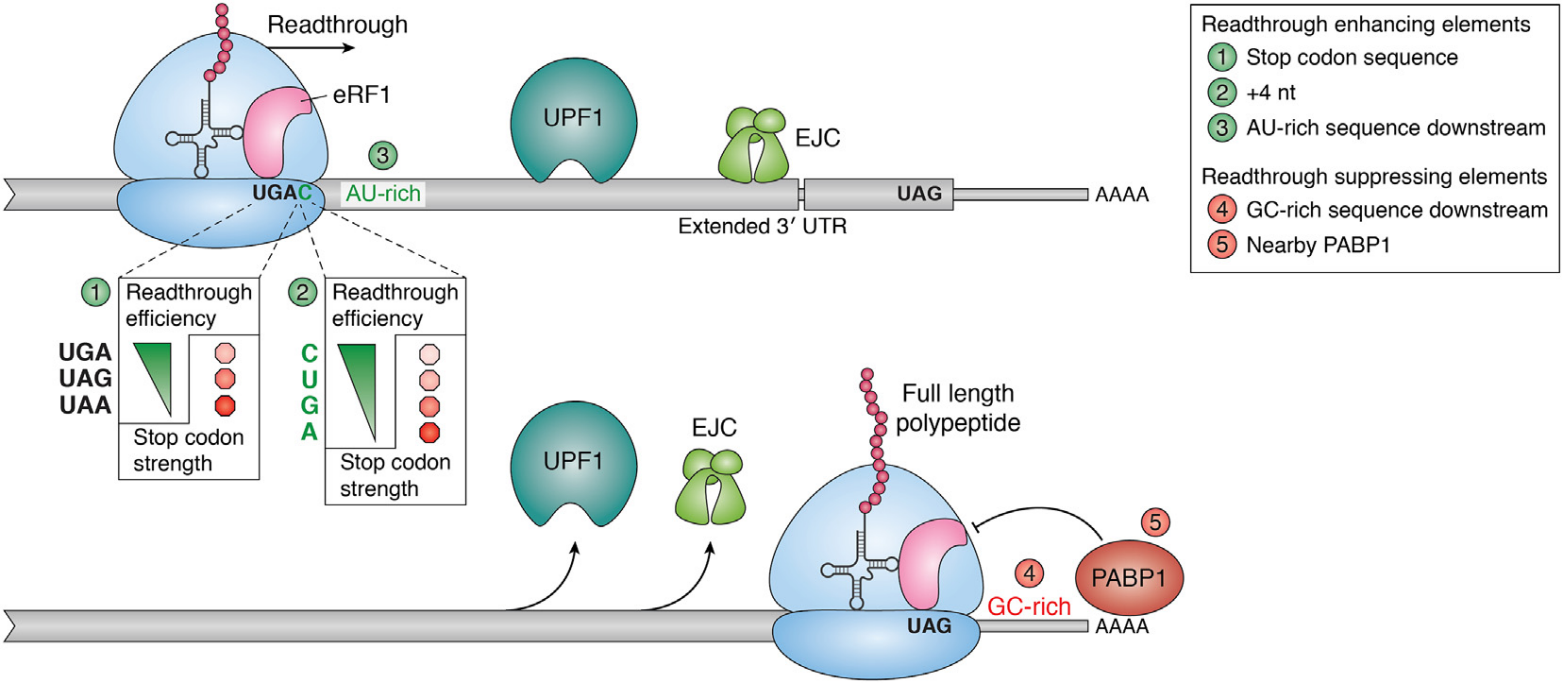
**Stop codon readthrough can prevent NMD.***Top*, factors that promote stop codon readthrough: (1) the stop codon triplet used, where the UGA codon is the most permissive stop codon, (2) the identity of the nucleotide at +4 position where stop codon readthrough is promoted the most by a C, and (3) an AU-rich region downstream of the stop codon. *Bottom*, factors that inhibit stop codon readthrough: These are typically found adjacent to the normal termination codons and include a GC-rich region immediately downstream of the stop codon and the presence of poly-A binding protein 1 (PABP1) in spatial proximity of the termination codon. NMD, nonsense-mediated mRNA decay.

When a ribosome reaches a stop codon, it may not always terminate translation. Instead, a noncognate aminoacyl-tRNA can outcompete eRF1 for recognition of stop codon in the A-site, thereby leading to continuation of translation into the downstream region. Such stop codon readthrough can occur naturally at any stop codon, albeit at an extremely low rate. However, certain sequence contexts can make ribosome readthrough more likely. As readthrough at stop codon yields an elongating rather than a terminating ribosome, leaky translation at stop codons antagonizes NMD (131). If ribosome translocation continues for extended distances past stop codons, this can further desensitize the mRNA for NMD by stripping off NMD activating factors such as UPF1 and EJC from downstream regions (Fig. 4). In *S. cerevisiae*, which does not rely on the EJC for NMD, a meager stop codon readthrough rate of 0.5%, or one out of every 200 ribosomes, can reduce NMD efficiency (131). Similarly, in mammalian cells, a readthrough rate of less than 1% is sufficient to displace the EJCs and/or UPF1 to inhibit NMD (49). The low amount of stop codon readthrough required to reduce NMD efficiency is among the many lines of evidence that supports NMD to occur during any round of translation (49, 131). If NMD was only triggered during the pioneer round of translation, a much higher rate of stop codon readthrough would be required to stabilize mRNA with PTCs.

#### Effect of stop codon context

Early genetic studies identified that various stop codon contexts can influence termination efficiency and hence ribosome readthrough rate (132). More recent genome-wide analyses of translational readthrough in both yeast and human cells have confirmed these previous observations (133, 134). The UGA stop codon is more permissive to readthrough than the other two stop codons (Fig. 4). The sequence surrounding the stop codon, both upstream (133, 135) and downstream (13, 131, 133, 136), can dramatically influence the rate of readthrough. The sequences immediately downstream of the stop codon have the strongest effect on readthrough rate. For instance, a cytosine immediately after the stop codon (at the +4 position) results in an increased readthrough rate across all eukaryotes tested (Fig. 4) (133, 134). As eRF1 also recognizes the +4 nt during translation termination leading to mRNA compaction (8, 13), it is conceivable that a cytosine at this position is least effective in stabilizing such a conformation, which could explain why UGAC is the most readthrough permissive stop codon context (133). An AU-rich sequence 15 nt upstream and downstream of stop codons also increases the rate of stop codon readthrough (133). The +4 nt and AU-rich regions downstream of the stop codon promote readthrough by preventing eRF1 from cleaving the peptidyl-tRNA bond (Fig. 4) (10). An earlier study suggested that potential base pairing between the readthrough promoting sequence and 18S rRNA may be responsible for altering termination efficiency (137). The penultimate codon can also affect readthrough frequency in *S. cerevisiae* (133, 135); the AUA, ACA, and GAC codons can enhance stop codon readthrough, whereas CGU, GCU, and UUA codons suppress it (134). There also exist sequence contexts that prevent readthrough and promote termination. For example, high GC content in regions immediately downstream of NTCs may have evolved to help prevent readthrough (Fig. 4) (133). When a ribosome terminates at a PTC introduced by a mutation or an error in the middle of a transcript, it is unlikely that such a GC-rich region is present immediately downstream, thus making PTCs more prone than NTCs to ribosome readthrough.

The effect of readthrough promoting sequence contexts on NMD is yet to be systematically evaluated, but the available anecdotal evidence supports the profound influence of increased readthrough on NMD efficiency (48, 138). In certain mammalian genes, a UGA stop codon followed by CUAG sequence promotes stop codon readthrough (139). When these readthrough-inducing stop codon contexts from the *OPRL1* and *AQP4* genes are introduced into an NMD reporter, it dramatically decreases reporter NMD efficiency in mammalian cells (46). Importantly, nonsense mutations in the *F9* gene, which cause hemophilia B because of inactivation of coagulation factor IX, lead to appreciable readthrough and only a mild phenotype when the nonsense mutation is in a readthrough permissible UUGAC context (140). Similarly, a nonsense mutation in the identical UUGAC context in *LAMA3*, which encodes laminin subunit a-3, causes increased readthrough, substantially increased full-length polypeptide expression, and less severe phenotype in a patient with junctional epidermolysis bullosa (141). A systematic analysis of potentially NMD-resistant nonsense variants also identified two other readthrough-prone human genes although the sequence context of these nonsense variants was not described (142).

A specialized stop codon readthrough mechanism is observed in the case of mRNAs encoding selenoproteins where “UGA” stop codons can be recoded as selenocysteine, a nonstandard amino acid (7). In this case, the selenocysteine recoding and readthrough is dictated by a *cis*-acting structured selenocysteine insertion sequence located in 3′ UTRs (7). In selenium-deficient conditions, translation terminates at the UGA “selenocysteine” codon and mRNA is degraded *via* NMD (7). Under selenium-replete conditions (in certain organs such as the liver), UGA is recoded as selenocysteine leading to readthrough and mRNA escape from NMD (7).

Specialized sequences that promote stop codon readthrough are also often employed by viruses to maximize the coding potential of their meager genetic resources (143, 144). Viral RNAs often use such a strategy to encode multiple polypeptides from the same viral mRNA. An example is the Moloney murine leukemia virus that uses translational recoding of stop codon to either produce Gag polyprotein or a Gag–Pol fusion polypeptide (143, 144). The viral reverse transcriptase, which is part of the Gag polyprotein, interacts with the C-terminal domain of eRF1 to prevent eRF1–eRF3 interaction and causes stop codon readthrough (143, 144). Other viral sequences that promote ribosome readthrough form stable RNA structures (144). Although the readthrough promoting viral sequences can lower NMD efficiency when inserted in NMD reporters (145), it is not clear if viruses use such strategies to specifically suppress NMD to promote their propagation. Still, such viral control elements offer an attractive tool to investigate the mechanisms of ribosome readthrough and its effect on NMD.

#### Influence of *trans*-acting factors

In addition to the sequence context surrounding the stop codon, disruption of the factors that promote translation termination can enhance readthrough. In different model systems, limited levels of release factors lead to reduced termination efficiency and increased ribosome readthrough (146, 147, 148). Under these conditions, readthrough can be even greater with a longer 3' UTR and hence lesser PABP1 interaction with the terminating ribosome (29, 134). This outcome is different from that under normal conditions when a reduced PABP1-terminating ribosome interaction would promote NMD and possibly reflects a different function of PABP1 during inefficient termination. Loss of other factors that enhance translation termination, such as Dbp5/Gle1 and their activator IP6, can also increase readthrough rates (34, 35, 36). Disruption of ribosome recycling, which will result in extended residence times of the terminated ribosome on a transcript, thereby increasing the chances of stop codon recognition by a noncognate aminoacyl-tRNA, also enhances ribosome readthrough. A reduction in levels of factors involved in ribosome splitting/60S subunit recycling, such as ABCE1 depletion in human cells and HCR1 disruption in yeast cells, leads to increased ribosome readthrough, and hence the removal of NMD activators from the 3′ UTRs and reduced NMD efficiency (40, 138, 149, 150). Similar outcomes are reported for the loss of 40S subunit recycling factors *TMA20* and *TMA22* in yeast cells (133, 149).

Disruption of NMD machinery also influences the amount of readthrough observed at PTCs. This observation supports a role for UPF factors in enhancing the efficiency of termination and/or ribosome recycling during premature termination. UPF1 knockout in *S. cerevisiae* increases stop codon readthrough rate by approximately twofold (131). Moreover, under these conditions, the difference in readthrough rates between readthrough permissive and standard stop codon contexts is much smaller than in wildtype, indicating an overall increase in readthrough (131). Thus, the relationship between UPF1 and stop codon readthrough is a reciprocal one. Just as ribosomes failing to terminate can displace UPF1, the presence of UPF1 can suppress stop codon readthrough (131). Importantly, the increased readthrough upon NMD inhibition in yeast can also be attributed to an indirect mechanism. Depletion of UPF factors in yeast stabilizes the PTC containing *ALR1* mRNA that encodes a magnesium transporter. The elevation of ALR1 levels in both NMD-deficient and NMD-proficient cells causes an increase in magnesium uptake, which leads to an increase in translational readthrough (151). Thus, the direct impact of NMD factors on outcomes of termination reaction in yeast and eukaryotes in general remains an important open question.

#### Therapeutic potential of ribosome readthrough

Readthrough of nonsense codons has long been recognized as a potential therapeutic strategy for diseases where a PTC leads to NMD and expression of a truncated protein. In such cases, promoting stop codon readthrough would allow for the production of a full-length protein that may retain wildtype function and ameliorate disease (142). A variety of small chemicals have been identified that can promote readthrough at stop codons. We refer readers to an excellent recent review for an in-depth discussion of such readthrough-promoting drugs, their mode of action, and various challenges in their clinical use (152). We want to highlight two key points here regarding these readthrough-promoting drugs. First, just like natural ribosomal readthrough, the drug-induced readthrough is likely to be influenced by stop codon sequence context, as was recently shown for the aminoglycoside class of antibiotics (133). Notably, aminoglycosides are the most widely investigated readthrough-promoting drugs that hold therapeutic potential in a wide variety of contexts, such as epilepsy (153), Duchenne muscular dystrophy (154), and cancer (155). Second, another readthrough-promoting chemical, PTC124 (also known as ataluren) that is already in clinical use for Duchenne muscular dystrophy treatment, was shown to specifically promote readthrough at PTCs but not NTCs (156). This observation also provides an argument in favor of potential mechanistic differences between normal and premature termination.

### Translation reinitiation following termination also silences NMD

**Figure 5:**
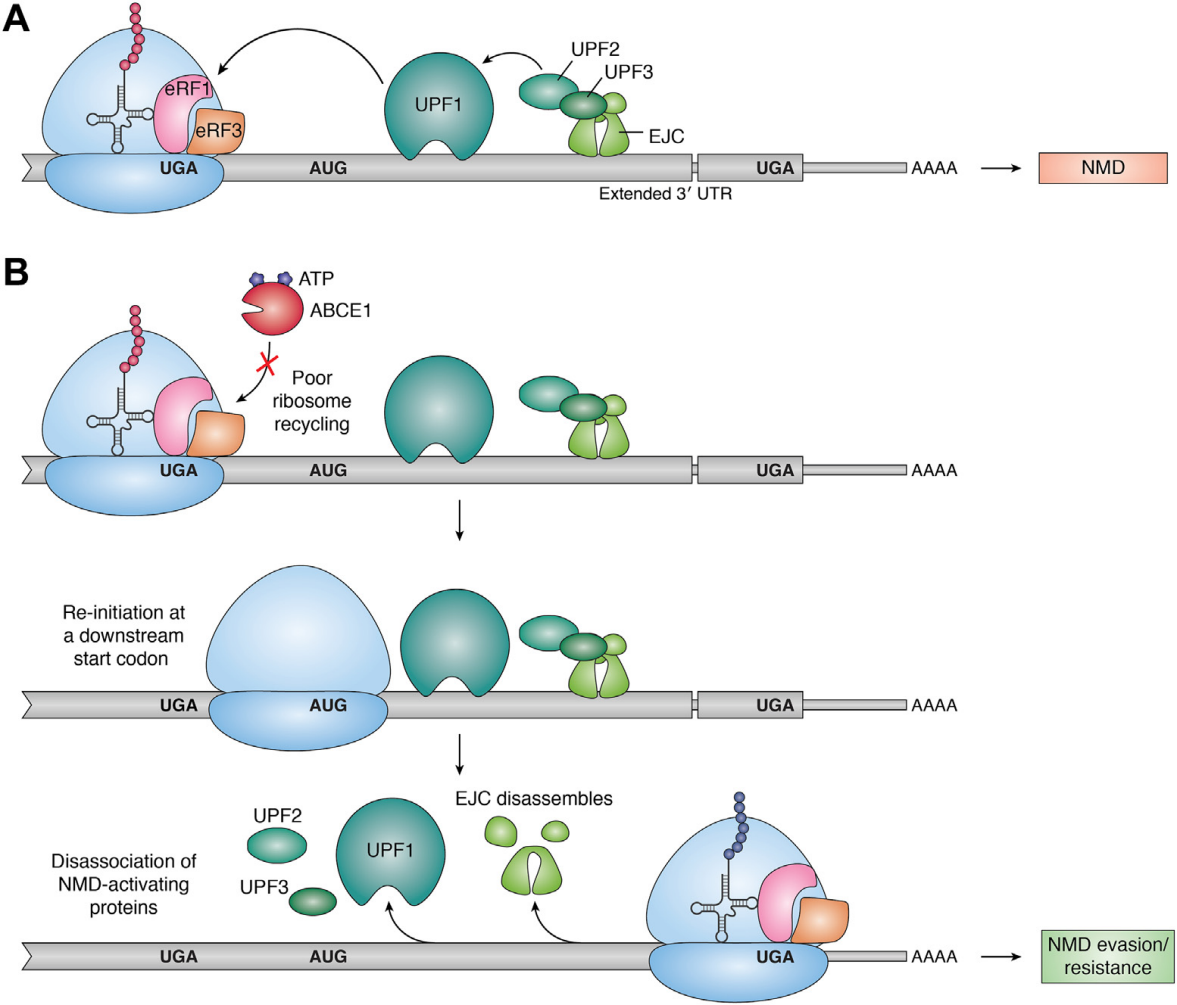
**Ribosome reinitiation after a PTC can prevent NMD.***A*, when the ribosome terminates at a PTC, it is recognized by downstream UPF1, with the aid of the EJC, and the transcript undergoes NMD. See Figure 2 for more details. *B*, when ribosome recycling occurs poorly at a premature termination codon, for example, because of limiting recycling factors like ABCE1, this can lead to the ribosome reinitiation at a nearby downstream start codon. Reinitiation causes displacement of NMD activating UPF and EJC proteins, escape from NMD and production of an N-terminally truncated or a unique polypeptide. EJC, exon junction complex; NMD, nonsense-mediated mRNA decay; PTC, premature termination codon.

Similar to stop codon readthrough is ribosome reinitiation, where a terminated ribosome restarts translation from a nearby AUG codon and into the 3′ UTR (52). Estimates from *S. cerevisiae* suggest that approximately 1 in 40 ribosomes can reinitiate at a downstream start codon after terminating at a nonsense codon (52). The impact of ribosome reinitiation is similar to readthrough as reinitiated ribosomes would translocate through sequences 3′ to PTCs, thereby dislodging NMD-activating proteins from these regions and making mRNAs resistant to NMD (Fig. 5). If reinitiation downstream of a PTC occurs from an in-frame start codon, it will produce an N-terminally truncated protein, which may or may not be beneficial (or even harmful) to the cell depending on the location of PTC. However, if the ribosome reinitiates in another frame, it will result in a novel polypeptide with little resemblance to the expected protein. It is also notable that translation reinitiation that causes NMD escape can also occur at noncanonical start codons downstream of a PTC although this happens at a lower rate (142, 157).

#### Factors that influence ribosome reinitiation

Translation reinitiation that interferes with NMD is observed more frequently when PTCs occur near normal start codons and when an AUG (methionine or Met) codon is also present in close vicinity of such stop codons. These general features are borne out of analysis of several disease-linked nonsense mutations that were found to be NMD refractory and from mutational studies using NMD reporters based on such genes. A triose phosphate isomerase gene–based reporter showed that PTCs within the first 30 nt of the triose phosphate isomerase coding sequence are resistant to downregulation *via* NMD, and this resistance is dependent on a nearby downstream AUG codon that exists in a favorable translation initiation context (112). Similar trends have been observed using reporters based on the β-globin gene wherein nonsense mutations cause β-thalassemia (158). Usually, nonsense mutations in β-globin result in NMD, but those near the 5′ end of the transcript (in exon 1) escape NMD (113, 159). Mutational analysis of β-globin reporters suggests that the NMD resistance of β-globin exon 1 nonsense mutations depends on translation reinitiation at a downstream in-frame AUG (Met55), thereby producing an N-terminally truncated protein (157). Translation reinitiation at downstream AUG codons also underlies NMD resistance of *TP53* (160) and α-globin (161) gene reporters, of multiple genes that encode N-terminally truncated proteins from their nonsense variants (142), and of several disease-causing nonsense alleles that escape NMD (see Refs. (162, 163, 164) for some examples). In certain diseases, such an escape from NMD can turn out to be beneficial. Nonsense mutations in the *ATRX* gene are embryonic lethal, but one mutation, R37X (a c.109C>T change in DNA sequence), avoids this fate (165). Reinitiation downstream of the PTC results in enough *ATRX* transcripts that survive NMD to produce a truncated but partly functional protein at ∼20% of the wildtype amount, which prevents the severe phenotype (165).

Recent large-scale analyses have further validated that reinitiation downstream of PTCs is one of the key factors for NMD resistance of an mRNA. An analysis of thousands of somatic nonsense mutations in the cancer genome atlas data verified that PTCs within the first 150 nt of coding sequence represent one of the three major classes of NMD evading features (48, 166, 167). Furthermore, while the rapid emergence of engineered nucleases has provided a facile tool to introduce nonsense/frameshift mutations to generate loss-of-function alleles, several studies have noted that such nonsense alleles often escape NMD (168). Reinitiation downstream of PTCs is one of the major reasons for this phenomenon (167, 168). Such a limitation can be circumvented by introducing NMD-inducing lesions far away from possible alternative translation initiation sites (169).

Ribosome reinitiation downstream of a stop codon can also be used to regulate endogenous transcripts *via* NMD. In a phenomenon observed in the *SRSF7* gene, a “split-ORF,” where the normal ORF is split by a PTC and a downstream start codon, is used to regulate SRSF7 levels (170). Translation reinitiation from the downstream start codon is required to remove the remaining EJCs from the second part of the ORF. Otherwise, mRNA suppression by NMD would not allow accumulation of the polypeptide encoded by the first half of the split-ORF that is required for SRSF7 regulation (170). Split-ORFs were reported to occur in a large number of human and mouse transcripts and proposed to be an important regulatory mechanism for RNA-binding proteins (170).

#### Possible mechanisms of ribosome reinitiation

How can a ribosome that has undergone termination reinitiate translation on the same mRNA? Although details of this process remain poorly understood, possible mechanisms have emerged from the investigation of mRNAs that contain uORFs, short translation regulatory ORFs in mRNA 5′UTRs that control ribosome access to the main ORF. uORFs have been well defined in stress response genes in yeast and mammalian cells (171), although their widespread prevalence in human and other genomes has been reported (171). Translation regulation by uORFs is achieved by controlling how frequently a ribosome will reinitiate translation on the start codon of the main/downstream ORF after it terminates on the uORF stop codon. Translation termination at uORF stop codons can also activate NMD as such a termination event is followed by a much longer “3′ UTR” that may even carry one or more EJCs. Investigations into factors that regulate translation reinitiation on the main ORF start codon have revealed that the short length of uORFs and short intercistronic distance between multiple ORFs are key factors that determine reinitiation efficiency (172, 173, 174, 175). Analysis of uORFs in yeast *GCN4* gene and human *ATF4* gene suggests that shorter distances traversed by ribosomes elongating in uORFs or scanning intercistronic sequences result in retention of certain protein factors such as eIF3B, a subunit of translation factor eIF3 with important roles in translation initiation, termination, and recycling (172, 176). Even core translation initiation factors eIF4E and eIF4G can remain associated with elongating 80S ribosomes for short distances (177) and can thereby explain how short uORFs can promote reinitiation more efficiently.

Reinitiation at a downstream start codon occurs in opposition of ribosome recycling (Fig. 5) (52). Thus, variations in ribosome recycling may be yet another factor that dictates reinitiation efficiency. For example, when ribosome splitting factor ABCE1 is depleted from yeast cells, ribosomes can reinitiate upstream or downstream of the stop codon, sometimes in a different reading frame (178). Similarly, knockdown of 40S recycling factors also increases the rate of reinitiation (39, 42, 43, 52).

#### Reinitiation-independent mechanisms of NMD escape?

Available evidence also suggests that other mechanisms may also dictate if an mRNA with an early stop codon is targeted to NMD. Romão *et al.* (27, 101) have proposed an alternative model for the NMD escape of β-globin exon 1 PTCs. According to their model, start codon proximal PTCs are juxtaposed with poly(A)-tail-bound PABPC1 because of mRNA circularization. The resulting PABPC1 interactions with the translation initiation and termination factors are proposed to cause termination at these PTCs to resemble normal termination and hence the escape from NMD. Furthermore, the potential for reinitiation may not always be enough to prevent NMD. A recent study has demonstrated that reinitiation following uORFs does not cause significant reduction in NMD efficiency (174). This investigation indicates that NMD induction efficiency of uORFs is more dependent on whether a uORF is translated or not and not by the degree of reinitiation following uORF termination. This evidence reinforces that regulation of NMD is a multilayered process where synergistic action of numerous factors that influence the NMD efficiency of a single transcript remains to be fully understood.

## Conclusion

The aforementioned discussion views the mechanism of NMD, a critical quality control and gene regulatory pathway, through the lens of translation termination. Various alternative outcomes during translation such as stop codon readthrough, reinitiation, and ribosome frameshifting complicates predictions whether NMD will occur on a particular transcript simply based on the presence of an NMD-inducing feature. Termination-linked *cis*- and *trans*-acting NMD modulators allow for transcript regulation to be a more nuanced process, capable of responding to cellular signals. At the same time, this flexibility also provides avenues for legitimate, even disease-causing, errors to escape the watchful gaze of NMD.

While the escape from NMD can lead to undesirable consequences such as diseases, they also have the potential for therapeutic targeting of the pathway. The understanding of the dynamics of translation termination and NMD can illuminate ways to counter the harmful effects of nonsense mutations. Both preventing escape from NMD or encouraging it in situations where producing an aberrant protein is the preferred response, have the potential to ameliorate the effects of a disease-causing mutation. To fully realize this potential, future work is needed to better understand the interactions between NMD factors and translation termination and recycling machinery. In particular, a more complete map of the interactions between ribosomes, termination factors, and NMD machinery would help in predicting how translation termination at abnormal stop codons will affect NMD.

## Conflict of interest

The authors declare that they have no conflicts of interest with the contents of this article.
